# Pigment Produced by Glycine-Stimulated *Macrophomina Phaseolina* Is a (−)-Botryodiplodin Reaction Product and the Basis for an In-Culture Assay for (−)-Botryodiplodin Production

**DOI:** 10.3390/pathogens11030280

**Published:** 2022-02-22

**Authors:** Sahib Alam, Hamed K. Abbas, Michael Sulyok, Vivek H. Khambhati, Wahab O. Okunowo, Wayne Thomas Shier

**Affiliations:** 1 Department of Medicinal Chemistry, College of Pharmacy, The University of Minnesota, Minneapolis, MN 55455, USA; dralam@aup.edu.pk (S.A.); wokunowo@unilag.edu.ng (W.O.O.); 2 Department of Agricultural Chemistry and Biochemistry, The University of Agriculture Peshawar, Peshawar 25130, Pakistan; 3 Biological Control of Pests Research Unit, Agricultural Research Service, U.S. Department of Agriculture, Stoneville, MS 38776, USA; vhk4@msstate.edu; 4 Department of Agrobiotechnology (IFA-Tulln), Institute of Bioanalytics and Agro-Metabolomics, University of Natural Resources and Life Sciences Vienna, Konrad Lorenzstr. 20, A-3430 Tulln, Austria; michael.sulyok@boku.ac.at; 5 Department of Biochemistry, College of Medicine, University of Lagos, Surulere 101017, Lagos State, Nigeria

**Keywords:** *Macrophomina phaseolina*, pigments, (−)-Botryodiplodin, charcoal rot disease, in-culture bioassay, amino acid, soybean, secondary metabolite, natural product, mycotoxin, phytotoxin, moniliformin, kojic acid

## Abstract

An isolate of *Macrophomina phaseolina* from muskmelons (*Cucumis melo*) was reported by Dunlap and Bruton to produce red pigment(s) in melons and in culture in the presence of added glycine, alanine, leucine, or asparagine in the medium, but not with some other amino acids and nitrogen-containing compounds. We explored the generality and mechanism of this pigment production response using pathogenic *M. phaseolina* isolates from soybean plants expressing symptoms of charcoal rot disease. A survey of 42 *M. phaseolina* isolates growing on Czapek-Dox agar medium supplemented with glycine confirmed pigment production by 71% of isolates at the optimal glycine concentration (10 g/L). Studies in this laboratory have demonstrated that some pathogenic isolates of *M. phaseolina* produce the mycotoxin (−)-botryodiplodin, which has been reported to react with amino acids, proteins, and other amines to produce red pigments. Time course studies showed a significant positive correlation between pigment and (−)-botryodiplodin production by selected *M. phaseolina* isolates with maximum production at seven to eight days. Pigments produced in agar culture medium supplemented with glycine, beta-alanine, or other amines exhibited similar UV-vis adsorption spectra as did pigments produced by (±)-botryodiplodin reacting in the same agar medium. In a separate study of 39 *M. phaseolina* isolates, red pigment production (OD_520_) on 10 g/L glycine-supplemented Czapek-Dox agar medium correlated significantly with (−)-botryodiplodin production (LC/MS analysis of culture filtrates) in parallel cultures on un-supplemented medium. These results support pigment production on glycine-supplemented agar medium as a simple and inexpensive in-culture method for detecting (−)-botryodiplodin production by *M. phaseolina* isolates.

## 1. Introduction

Red pigment production by halophilic bacteria on salted herring fish was historically recognized as a sign of spoilage. Microbial pigment production continues to have a substantial economic impact by reducing the suitability of agricultural products for sale and use. Probably the most important single example in the US at present is blue stain in wood products caused by *Ophiostoma* spp. [1]. Microbial pigment production in association with fruit spoilage also remains an ongoing problem. Dunlap and Bruton [2] reported that muskmelons (*Cucumis melo*) infected by *Macrophomina phaseolina* contained a red pigment that was also produced in liquid cultures by the isolate, TX-33, when the medium was supplemented with glycine, alanine, leucine and asparagine. A number of other amino acids and nitrogen containing compounds failed to support pigment synthesis. 

The soil-borne fungus *Macrophomina phaseolina* (Tassi) Goidanich [3], infects over 500 host plants, including commercially important food and fiber producing row crops, trees and ornamentals in the United States and around the world [4,5,6]. The fungus causes a variety of plant diseases, including summer wilt, dry weather wilt, and black root. Of particular concern is charcoal rot of soybean, which is currently one of the major causes of crop yield loss and seed quality deterioration in southern United States [7,8,9,10,11,12,13]. *M. phaseolina* overwinters as microsclerotia in the soil reservoir or in roots or stems of crop residues and can survive harsh weather conditions for up to three to five years in the soil [14,15]. Disease development is typically favored by factors that cause plant stress, so that countries with low moisture, high temperatures (30–35 °C), poor soil fertility, and high seeding rates usually have more severe cases of disease caused by *M. phaseolina* [6,15,16]. Climate change is expected to result in hotter and drier conditions in most parts of the world [17], and these are climatic conditions that favor increased incidence and severity of plant disease caused by *M. phaseolina* [4]. 

*M. phaseolina* has been reported to produce more than a dozen mycotoxins and other secondary metabolites [18], including phaseolinone, phaseolinic acid and (−)-botryodiplodin [19,20,21,22,23,24]. (−)-Botryodiplodin is a simple ribose analog mycotoxin produced by several species of fungi, including *Lasiodiplodia theobromae* (Pat.) Griffon & Maubl. [syn. *Botryodiplodia theobromae* Pat.] [25], *Talaromyces coalescens* (Quintan.) Samson, Yilmaz & Frisvad [syn. *Penicillium coalescens* Quintan.] [26], *Penicillium roqueforti* Sopp [27,28], *Talaromyces stipitatus* Stolk & Samson [syn. *Penicillium stipitatum* Thom] [29], *M. phaseolina* [21] and others [30]. This mycotoxin has received attention due to its potent antibiotic [31], anticancer [32] mutagenic, cytotoxic [33] and phytotoxic activities [34], as well as the ability to induce protein-DNA crosslinking in mammalian cells [35,36]. Among (−)-botryodiplodin producing fungi, *P. roqueforti* is a common contaminant of processed food [28]. (−)-Botryodiplodin may also play a role in the initial stages of root infection by *M. phaseolina* in which it kills root tissue creating necrotic areas through which the fungus can readily penetrate [34]. 

Methods have been developed to measure (−)-botryodiplodin contamination of foods and feeds [18], but they are not practical for screening every lot of foods and feeds, especially in developing countries, due to the expense and lack of required equipment. This situation has created interest in developing in-culture methods as a prescreen to identify agricultural product lots that are the candidates for the export market. Additionally, there has been interest in developing and using culture methods for detecting non-toxigenic fungal isolates for potential use as biocontrol agents. For example, pigment formation by *Aspergillus flavus* in culture media has been used as a tool for developing cultural methods to detect toxin production and identify non-toxigenic strains [37,38]. (−)-Botryodiplodin has been reported to slowly form a pink to red stain on human skin when the purified toxin is applied to it [25,32]. The pigment forms on the cornified outer layer of the skin, but not on protein-free surfaces to which (−)-botryodiplodin is applied [39]. The pigment formed on the surface of human skin exposed to (−)-botryodiplodin cannot be solubilized with water or a variety of organic solvents, consistent with formation of pigment molecule(s) attached to part of skin protein molecules through a covalent bond. These observations led to the hypothesis that pigment production by *M. phaseolina* in culture may be the result of production of (−)-botryodiplodin, which then reacts with amino group-containing substances in the culture medium to form the actual pigments. In this paper we report the results of a series of studies that serve to evaluate the hypothesis that pigment production by pathogenic isolates of *M. phaseolina* in the presence of glycine reflects the production of the mycotoxin (−)-botryodiplodin, which then reacts with the glycine to produce the actual pigment. We demonstrate that the pigment produced is a reaction product with (−)-botryodiplodin, and we present evidence that the phenomenon can be adapted into the first reported in-culture method for detecting (−)-botryodiplodin-producing ability utilizing glycine supplementation of the culture medium. 

## 2. Results

### 2.1. Selection of M. phaseolina Isolates for Detailed Study

**Figure 1:**
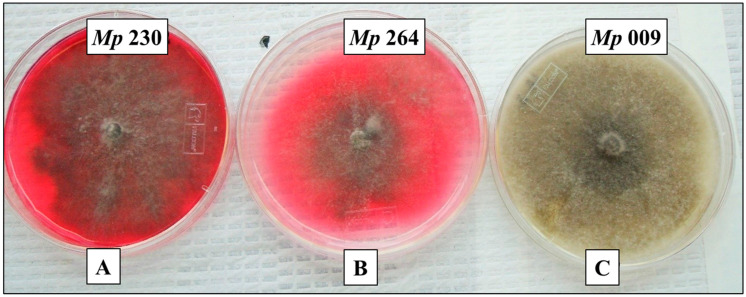
Cultures of three pathogenic *M. phaseolina* isolates that illustrate the three levels of pigment production observed within a collection of 42 isolates from soybean plants exhibiting symptoms of charcoal rot, specifically high levels of pigment production by isolate *Mp* 230 (**A**), moderate levels of pigment production by isolate *Mp* 264 (**B**), and no detectable pigment by isolate *Mp* 009 (**C**).

Production of red pigment when cultured on Czapek-Dox agar medium supplemented with 10 g/L glycine (Figure 1) was observed visually for 71% of isolates in a collection of 42 *M. phaseolina* isolates from soybean plants exhibiting charcoal rot in Mississippi. Pigment production by *M. phaseolina* isolates fell approximately into the following three categories: high levels of pigment (e.g., *M. phaseolina* isolates *Mp* 183 and *Mp* 230), moderate levels of pigment (e.g., *M. phaseolina* isolates *Mp* 264 and *Mp* 312), and no pigment (e.g., *M. phaseolina* isolates *Mp* 009 and *Mp* 239). In previous studies [24], (−)-botryodiplodin production by pathogenic *M. phaseolina* isolates *Mp* 009 and *Mp* 239 was below the limit of detection in culture filtrates, but for both isolates the culture filtrates contained substantial toxicity in soybean leaf disc cultures, consistent with these isolates producing different, as yet unidentified toxin(s).

### 2.2. Optimum Glycine Concentration for Pigment Production

**Figure 2:**
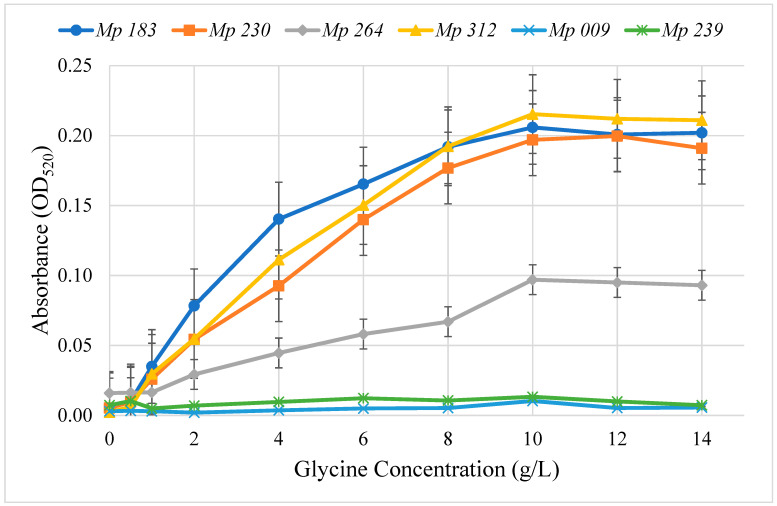
Effect of glycine concentration added to Czapek-Dox agar medium on pigment formation by selected *M. phaseolina* isolates. Pigment levels were measured in triplicate in extracts from agar plugs cut in the culture medium at the indicated times determined as OD_520_. Isolates *Mp* 183, *Mp* 230, *Mp* 264 and *Mp* 312 produced significantly (ANOVA, *p* < 0.001) greater amounts of pigment than *Mp* 009 and *Mp* 239 at all glycine concentrations greater that 1 g/L.

The concentration dependence on glycine for pigment production on Czapek Dox agar medium supplemented with glycine was determined for the six selected isolates that produced pigment (Figure 2). Pigment production was quantitated by extracting it from 4 mm diameter agar plugs cut from plates containing a range of glycine concentrations and measuring absorbance at the λ_max_, 520 nm. The highest level of pigment production for all isolates was achieved at 10 mg/mL of glycine, after which no further increase was noted in the pigment intensity. Pigment production by *M. phaseolina* isolates at 10 g/L glycine was *Mp* 312 > *Mp* 183 > *Mp* 230 > *Mp* 264, which was significantly (ANOVA, *p* < 0.001) greater at all glycine concentrations over 1 g/L than OD_520_ absorbance by the non-pigment producers *Mp* 009 and *Mp* 239. Extracts from *Mp* 009 and *Mp* 239 contained only background OD_520_ that did not increase with increasing glycine concentrations. None of the six selected *M. phaseolina* isolates showed any significant effect by glycine (0 to 14 g/L) added to Czapek-Dox agar medium on growth rate measured as the increase in colony diameter (data not shown). 

### 2.3. Time Course for Pigment Production by Selected Isolates

**Figure 3:**
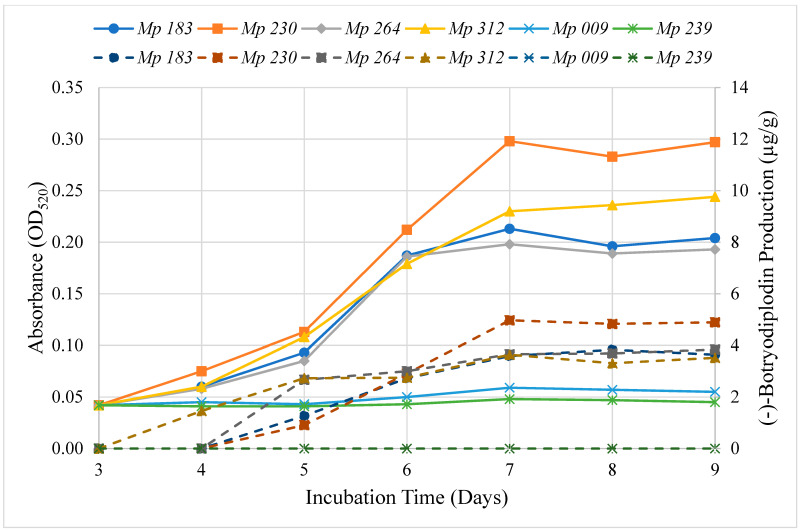
Time course of the production of pigment (solid lines) and of (−)-botryodiplodin (dashed lines) on Czapek Dox agar medium supplemented with glycine (10 g/L) by selected isolates of *M. phaseolina*, including two high level pigment producers, *Mp* 183 (●) and *Mp* 230 (■); two moderate level pigment producers, *Mp* 264 (♦) and *Mp* 312 (▲) and two pigment non-producers, *Mp* 009 (x) and *Mp* 239 (∗). Pigment and (−)-botryodiplodin levels were measured in extracts from agar plugs cut in the culture medium at the indicated times. Pigment levels were determined as OD_520_ of pooled triplicate aqueous extracts and (−)-botryodiplodin levels in pooled triplicate ethyl acetate extracts by LC-MS. The rate of pigment production by the selected *M. phaseolina* isolates correlated with the rate of (−)-botryodiplodin production in a highly significant manner (*r* = 0.96, *p* ≤ 0.0001, regression analysis).

The time course of extractable pigment production by the six selected isolates was determined on Czapek-Dox agar media supplemented with 10 g/L glycine (Figure 3). Extracted material absorbing at 520 nm was first observed at three days of incubation and increased in intensity until day seven, after which no further increase was noted in the intensity of color produced by the selected isolates. The time course study confirmed the absence of pigment production by *Mp* 009 and *Mp* 239 and the high level of pigment production by *Mp* 183 and *Mp* 230, but not the intermediate level of production by *Mp* 312, which exhibited a high level of pigment production. 

### 2.4. Effect of Czapek-Dox Medium Components on Pigment Production by Selected Isolates of M. phaseolina

**Figure 4:**
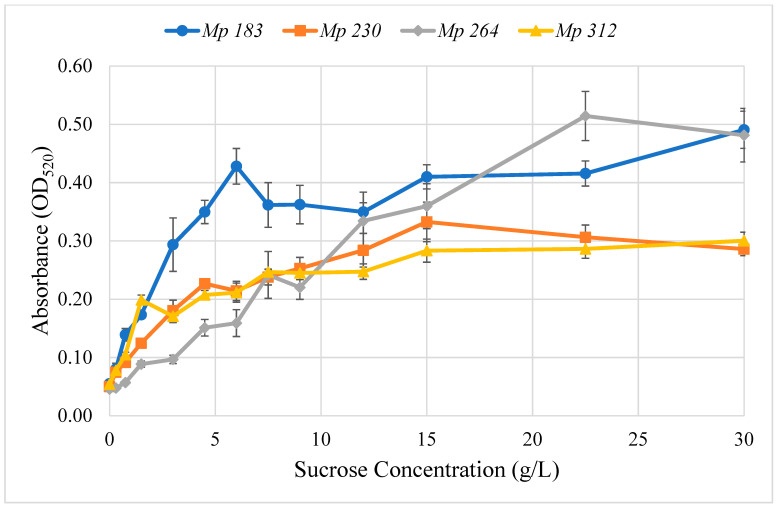
Effect of adding sucrose to modified Czapek-Dox agar medium prepared without sucrose on red pigment production by four selected pigment-producing *M. phaseolina* isolates, *Mp* 183 (-●-), *Mp* 230 (-■-), *Mp* 264 (-♦-) and *Mp* 312 (-▲-). The optimum sucrose concentration varied with *M. phaseolina* isolate, but all exhibited optimum red pigment production at the sucrose concentration used in conventional Czapek-Dox agar medium (30 g/L).

Various mechanisms for regulating pigment production by *M. phaseolina* isolates are plausible. One hypothetical regulatory mechanism that was investigated was that pigment production was stimulated in a differential manner by a culture medium component. This hypothesis was readily tested with Czapek-Dox agar medium, which has a defined composition, by inoculating culture media prepared with one component selectively deleted, except glycine and agar, for which the requirement or lack thereof are known from the studies of Dunlap and Bruton [2]. Among Czapek-Dox medium components, only sucrose stimulated pigment production by the four selected pigment-competent *M. phaseolina* isolates growing on agar plugs of complete Czapek-Dox medium, when the deleted component was added back in a well cut in agar medium. The concentration dependence of pigment production by sucrose in Czapek-Dox agar medium by selected *M. phaseolina* isolates was determined (Figure 4). All tested isolates increased pigment production with increasing sucrose concentration, which then plateaued above 15 g/L. Half-maximal pigment stimulation was observed in the range of 2–7 g/L sucrose. 

### 2.5. Pigment Production by Reaction of (±)-Botryodiplodin with Glycine and Other Amines 

**Figure 5:**
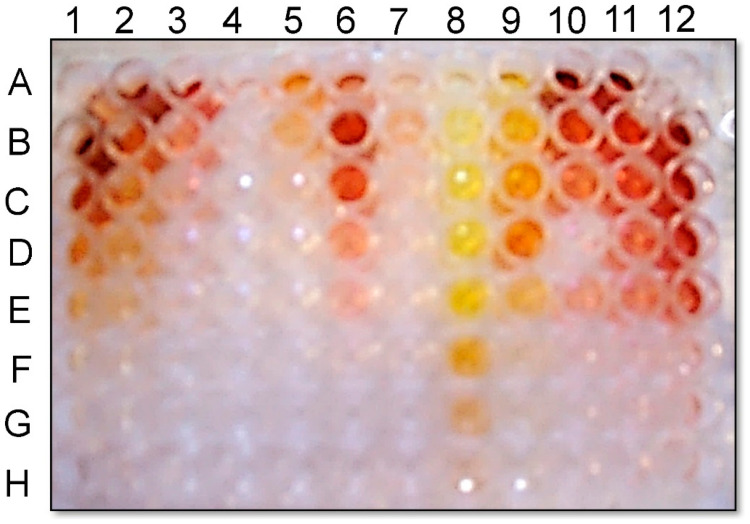
Pigment formation by reaction of (±)-botryodiplodin solution with various amines in the presence of air. Pigment production by reaction of (±)-botryodiplodin (0.5 mg/mL) and the following amines at the indicated initial concentrations and at serial 2-fold, 2-fold then 2.5-fold dilutions in the column below it: column 1: concentrated ammonium hydroxide, 20% (*v*/*v*); column 2: ammonium chloride, 10 mg/mL; column 3: poly-L-lysine hydrobromide, 1 mg/mL; column 4: bovine serum albumin, 1 mg/mL; column 5: chitosan hydrochloride, 1 mg/mL; column 6: ethylamine, 10 mg/mL; column 7: hexylamine, 10 mg/mL; column 8: ethylenediamine, 10 mg/mL; column 9: ethanolamine, 10 mg/mL; column 10: methylamine hydrochloride, 10 mg/mL; column 11: L-lysine dihydrochloride, 10 mg/mL; column 12: glycine 10 mg/mL.

Serial dilutions of glycine and a series of other amines in water were examined for pigment production with added (±)-botryodiplodin [40] in air. Red, orange, or yellow pigments were observed (Figure 5) with the most intense pigmentation being observed for glycine and β-alanine supplementation. When the reactions were run in degassed water in a nitrogen atmosphere, greatly reduced or no pigmentation was produced by (±)-botryodiplodin reaction with the various amines.

### 2.6. Mycotoxin Production by the Selected Isolates of M. phaseolina

**Figure 6:**
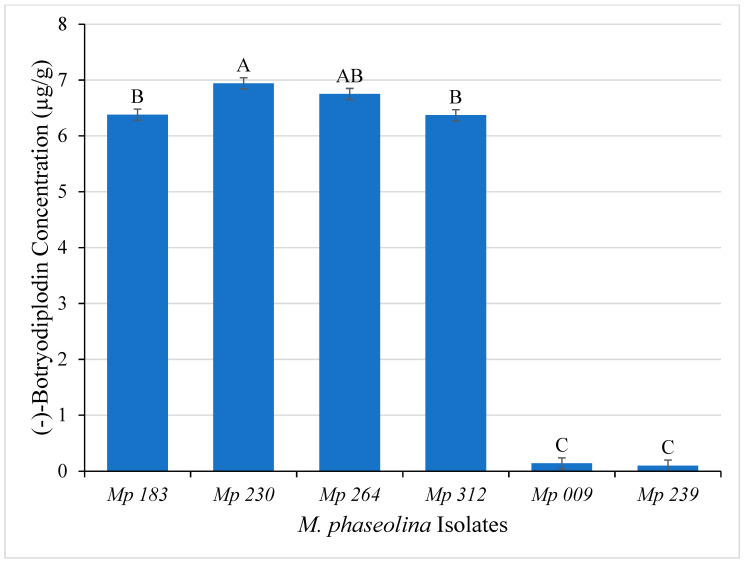
(−)-Botryodiplodin production (µg/g) by selected *M. phaseolina* isolates cultured on Czapek-Dox agar medium (means ± standard deviation). Values with the same letter are not significantly different (ANOVA, *p* < 0.05).

Initial studies to evaluate the hypothesis that pigment produced by *M. phaseolina* in culture results from producing (−)-botryodiplodin that reacts with glycine were carried out using LC-MS analysis of ethyl acetate extracts of Czapek-Dox agar culture medium of the six selected *M. phaseolina* isolates. The pigment producing isolates (*Mp* 183, *Mp* 230, *Mp* 264 and *Mp* 312) produced significantly (ANOVA, *p* ≤ 0.05) greater amounts of (−)-botryodiplodin after seven days of incubation than did the non-pigment producers (*Mp* 009 and *Mp* 239) (Figure 6). The highest amounts of (−)-botryodiplodin were produced by the isolate *Mp* 230 (6.94 µg/g) followed by *Mp* 264 (6.75 µg/g), whereas the lowest amounts were produced by non-pigment producing isolates *Mp* 009 (0.01 µg/g) and *Mp* 239 (0.02 µg/g).

The six selected *M. phaseolina* isolates were also examined using an LC-MS/MS system capable of identifying and quantitating production of >800 known fungal metabolites for production by isolates cultured in Czapek-Dox broth medium (Table 1). The selected six *M. phaseolina* pathogenic isolates produced many of the 15 metabolites identified previously [18]. The following known fungal metabolites were not detected as being produced by any of the *M. phaseolina* isolates studied: 3-nitropropionic acid, brevianamide F, citrinin, emodin, endocrocin, ethylorsellinic acid, infectopyron, mellein, methylorsellinic acid, monocerin, and N-benzoyl-phenylalanine. Production of (−)-botryodiplodin confirmed that *M. phaseolina* isolates that produce pigment on agar medium in the presence of glycine produced substantial levels of (−)-botryodiplodin, whereas *M. phaseolina* isolates that did not produce pigment, did not produce (−)-botryodiplodin above the limit of detection. Production of two metabolites not previously reported to be produced by *M. phaseolina* [18], cyclo(L-Pro-L-Val) and dihydrocitrinone, were produced by *M. phaseolina* isolates that did not produce (−)-botryodiplodin nor pigment. None of the other major metabolites produced by the selected *M. phaseolina* isolates (kojic acid, moniliformin, orsellinic acid) produced pigment by reaction overnight in air with glycine in aqueous solution at 10 g/L (data not shown). 

### 2.7. Time Course for (−)-Botryodiplodin Production by Selected Isolates

(−)-Botryodiplodin production by the selected isolates of *M. phaseolina* growing on Czapek-Dox agar medium was determined during an incubation period of nine days (Figure 3). The pigment producing *M. phaseolina* selected isolates (*Mp* 183, *Mp* 230, *Mp* 312 and *Mp* 264) all produced (−)-botryodiplodin beginning on day four, peaking on day seven and plateauing after that, and the non-pigment producing *M. phaseolina* selected isolates (*Mp* 009 and *Mp* 239) also did not produce (−)-botryodiplodin at measurable levels above background. There was a highly significant positive correlation between the rate of pigment production and of (−)-botryodiplodin production (*r* = 0.96, *p* ≤ 0.0001, regression analysis) for the selected *M. phaseolina* isolates. 

### 2.8. Absorption Spectra of Pigments Produced by Selected M. phaseolina Isolates and Pigments Formed by Reaction with (±)-Botryodiplodin in Czapek-Dox Agar Medium with Glycine

**Figure 7:**
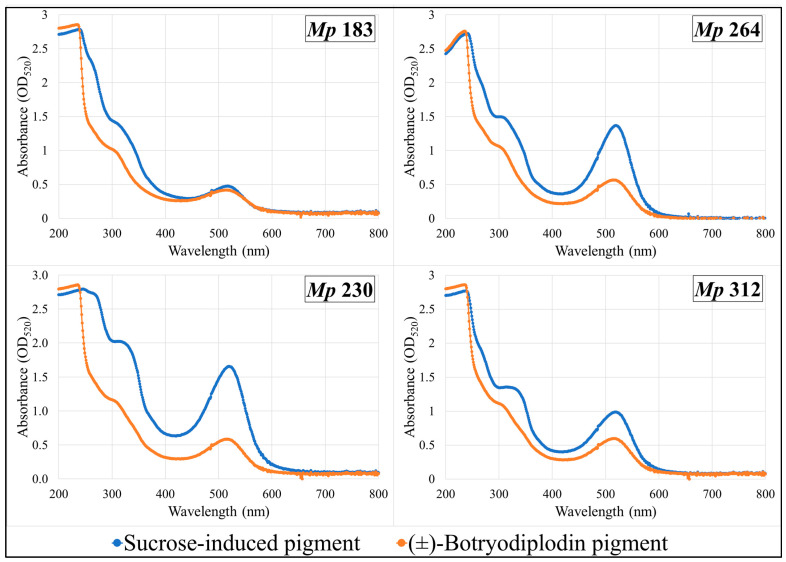
Ultraviolet-visible absorption spectra of pigments formed by reaction of glycine with synthetic (±)-botryodiplodin and with the sucrose-stimulated mycotoxin released from selected *Macrophomina phaseolina* (*Mp*) isolates.

**Figure 8:**
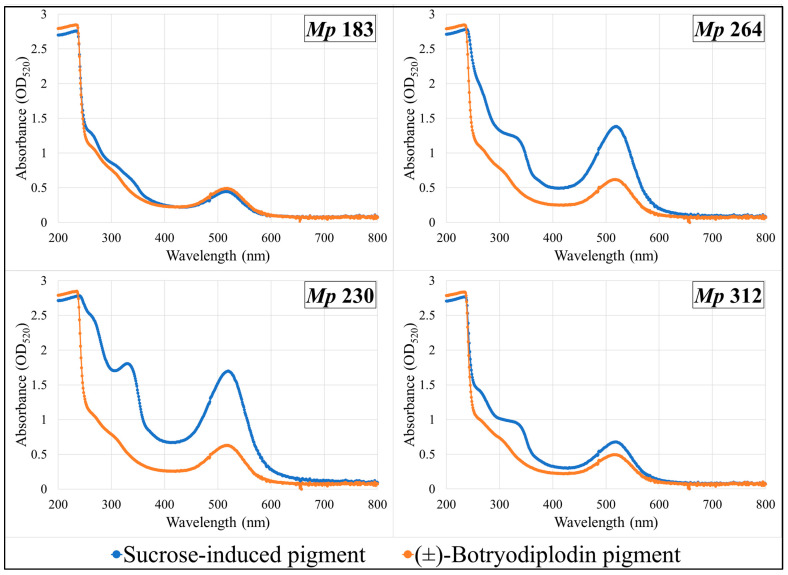
Ultraviolet-visible absorption spectra of pigments formed by reaction of β-alanine with synthetic (±)-botryodiplodin and with the sucrose-stimulated mycotoxin released from selected *Macrophomina phaseolina* (*Mp*) isolates.

Pigments produced by the four selected pigment-producing *M. phaseolina* isolates (*Mp* 183, *Mp* 230, *Mp* 312, and *Mp* 264) growing on Czapek-Dox agar medium lacking sucrose supplemented with glycine and other amines (β-alanine, lysine hydrochloride, ethylamine hydrochloride, or ethylenediamine dihydrochloride) were extracted and compared by their UV-vis absorbance spectra with pigment formed in the same plates by reaction with synthetic (±)-botryodiplodin added to a well cut in the agar layer. UV-vis absorption spectra of extracts from plates supplemented with glycine (Figure 7) or with β-alanine (Figure 8) are shown. Glycine and β-alanine both resulted in stronger absorption and more bands than with amines lacking nearby double bonds. The UV-vis spectra for pigment extracts contained the same absorbance bands for sucrose-induced fungal pigment as for pigment produced by reaction with (±)-botryodiplodin for each of the four *M. phaseolina* isolates with each of the five amines. 

### 2.9. Pigment Production on Glycine-Supplemented Medium As an In-Culture Method for Demonstrating (−)-Botryodiplodin Producing Ability by Pathogenic Isolates of M. phaseolina

**Figure 9:**
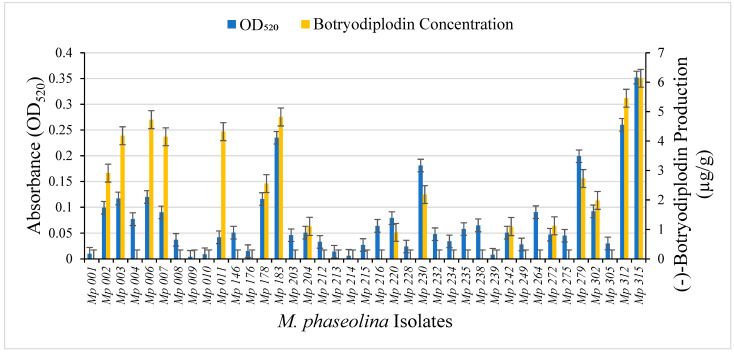
Production by the indicated pathogenic isolates of *M. phaseolina* from soybean plants expressing symptoms of charcoal rot disease of (i) red pigment (blue bars on the left) measured as light absorbance at 520 nm in aqueous extracts from Czapek-Dox agar culture medium supplemented with glycine (10 g/L); and (ii) (−)-botryodiplodin (orange bars on the right) measured by LC-MS (µg/g) in ethyl acetate extracts from parallel cultures on Czapek-Dox agar medium with no glycine. Red pigment production by the *M. phaseolina* isolates in the collection cultured on glycine-supplemented medium exhibited a highly significant positive correlation (*rs* = 0.705, *p* ≤ 0.0001, Spearman’s rank correlation test) with (−)-botryodiplodin production on the same medium without glycine.

A collection of 39 pathogenic isolates of *M. phaseolina* from soybean plants expressing symptoms of charcoal rot were cultured in triplicate on Czapek-Dox agar medium with added glycine (10 g/L) for seven days at room temperature (22–24 °C). For each, pigment production was determined by measuring absorption at 520 nm in aqueous extracts from triplicate agar plugs and (−)-botryodiplodin production was determined by LC-MS analysis of ethyl acetate extracts from triplicate agar plugs (Figure 9). The *M. phaseolina* isolates that did not produce pigment (*Mp* 009 and *Mp* 239) also did not produce (−)-botryodiplodin at measurable levels above background. There was a highly significant positive correlation between pigment production and (−)-botryodiplodin production (*rs* = 0.705, *p* ≤ 0.0001, Spearman’s rank correlation test) for the collection of *M. phaseolina* isolates. For example, the isolate (*Mp* 315) that produced the largest amount of pigment (OD_520_ = 0.349) also produced the highest amount of (−)-botryodiplodin (6.134 µg/g), whereas the isolate (*Mp* 009) with the lowest absorbance (OD_520_ = 0.005) did not produce a detectable amount of (−)-botryodiplodin. 

## 3. Discussion

The observation of Dunlap and Bruton [2] that an isolate of *M. phaseolina* could produce red pigment when cultured in medium containing glycine and other amines was confirmed in two approaches using *M. phaseolina* isolates from soybean plants exhibiting charcoal rot in Mississippi when the isolates were grown on glycine-supplemented Czapek-Dox agar medium. In the first study, red pigment production was observed visually in 71% of the isolates in a 42-isolate collection. In the second study, red pigment production was quantitated by light absorption at 520 nm in aqueous extracts prepared from the agar medium. Red pigment production, as indicated by OD_520_ ≥ 0.02 in extracts, was observed for 82% of an overlapping group of 39 *M. phaseolina* isolates. While the phenomenon reported by Dunlap and Bruton [2] is common in pathogenic isolates of *M. phaseolina* from soybean, it is not a universal characteristic of the fungus. 

The six pathogenic *M. phaseolina* isolates selected for more detailed study were shown (Table 1) to produce many of the metabolites first identified as being produced by *M. phaseolina* in a previous study [18]. The two pathogenic *M. phaseolina* isolates that did not produce red pigment or (−)-botryodiplodin in this study, *Mp* 009 and *Mp* 239, were shown previously [24] to produce culture filtrates in which (−)-botryodiplodin was below the limit of detection, but with both isolates the culture filtrates contained substantial toxicity in soybean leaf disc cultures, consistent with these isolates producing different, as yet unidentified toxin(s). In the present study, production of two metabolites not previously reported to be produced by *M. phaseolina* [18], cyclo(L-Pro-L-Val) and dihydrocitrinone, were shown (Table 1) to be produced by *Mp* 009 and *Mp* 239.

In-culture pigment production provides a useful tool for experimentally examining regulation of (−)-botryodiplodin production and its role in the root infection process of pathogenic *M. phaseolina*. It is not obvious how the observed stimulation of pigment and (−)-botryodiplodin production by sucrose could be involved in the initial infection of roots from the soil reservoir, but the experimental system used in these studies is amenable for use in studying other root-released substances for a potential role in mediating root infection. However, simulation of (−)-botryodiplodin release by high concentrations of sucrose does provide a plausible mechanism for *M. phaseolina* hyphae to exit the root systems of extensively infected plants in order to migrate through soil and infect interdigitating roots of neighboring plants. 

In-culture assays for the ability of fungi to produce mycotoxins have a variety of uses. For example, the methods of Lin and Dianese [41] and of Saito and Machida [42], which detect unutilized aflatoxin biosynthetic intermediates, have been useful in screening large numbers of isolates in studies such as examining the role of toxigenicity in the fungal life cycle and for the initial selection of atoxigenic isolates as potential biological control agents [43]. The in-culture assay for (−)-botryodiplodin production developed in this study is also potentially useable for identifying non-pathogenic biocontrol strains of *M. phaseolina* that might be used to treat field soil to prevent charcoal rot and other plant diseases caused by the fungus. Techniques employed in these studies correlating pigment and mycotoxin concentrations in extracts from plugs cut from agar culture medium suggest the potential to develop quantitative in-culture methods for measuring mycotoxin production by fungal isolates. Quantitative measurement of (−)-botryodiplodin production is possible, since the pigment being measured is formed from the toxin. Another example is aflatoxin measured by fluorescence in the presence of β-cyclodextrin [44]. Quantitative measurement of toxin production is not possible for in-culture assays in which the measured pigmentation is indirectly related to toxin production, such as from unutilized biosynthetic intermediates [41,42,45]. 

The results of these studies support the conclusion that pigment production by isolates of *M. phaseolina* in the presence of glycine and other amines, as reported by Dunlap and Bruton [2], results from production of the mycotoxin (−)-botryodiplodin, which then reacts with glycine or other amines to produce pigment. This conclusion was supported by two independent types of studies correlating red pigment production with (−)-botryodiplodin production, namely a correlation between rates of production of red pigment and of (−)-botryodiplodin in four of the *M. phaseolina* isolates selected for more intensive study (Figure 3) and a correlation between levels of red pigment and (−)-botryodiplodin production by a collection of 39 pathogenic *M. phaseolina* isolates (Figure 9). This conclusion implies that no related pigment-production genes are expected to be present in the *M. phaseolina* genome in either cassette or dispersed form. However, genes for (−)-botryodiplodin-producing enzymes are expected to be present in the genome of pigment-producing isolates of *M. phaseolina*. The absence of pigment production by some *M. phaseolina* isolates, such as *Mp* 009 and *Mp* 239, may be due to the absence of functional genes for (−)-botryodiplodin-producing enzymes, or the existence of a regulatory process that turns off expression. Additional studies are needed to determine the basis for atoxigenicity in *M. phaseolina* isolates.

## 4. Conclusions

The majority of pathogenic isolates of *M. phaseolina* cultured from soybean plants exhibiting symptoms of charcoal rot disease produced red pigment when cultured on Czapek-Dox agar medium supplemented with glycine, confirming a report by Dunlap and Bruton [2]. *M. phaseolina* isolates that produced red pigment were also observed to produce the mycotoxin (−)-botryodiplodin, which then reacted with glycine in the presence of air to indirectly produce the pigment, rather than being direct producers of the pigment as a secondary metabolite. When the reaction mixture is degassed and stored under nitrogen, little or no pigment is produced. Treatment with sodium cyanoborohydride (Borch reduction) resulted in near complete loss of pigmentation, consistent with air oxidation being needed to produce a Schiff base that extends conjugation in the pigment structure. Production of pigment and of (−)-botryodiplodin are both stimulated by sucrose at the concentration present in Czapek-Dox medium. Selected *M. phaseolina* isolates that were studied in more detail produce various mycotoxins and other metabolites, but only (−)-botryodiplodin produces red pigment by reacting with amines in solution. Pigments produced by sucrose-stimulated *M. phaseolina* isolates have the same UV-vis absorbance spectral wavelength maxima as pigments produced by direct reaction of (±)-botryodiplodin with glycine and other amines added to the culture medium. Pigment production in the presence of 10 g/L glycine in Czapek-Dox agar medium correlates significantly with the time course for (−)-botryodiplodin production by selected *M. phaseolina* isolates and for total (−)-botryodiplodin production after seven days in culture for a collection of 39 *M. phaseolina* isolates from soybean plants exhibiting symptoms of charcoal rot disease. The latter experiment serves as a validation of pigment production in glycine supplemented Czapek-Dox agar medium as an in-culture assay for ability to produce (−)-botryodiplodin. An in-culture assay for (−)-botryodiplodin production is a potentially useful research tool in areas such as studies on mechanisms controlling toxin production in pathogenic strains of *M. phaseolina*, the use of (−)-botryodiplodin in root infection mechanisms and the selection of non-producing *M. phaseolina* strains for evaluation as biocontrol agents to prevent charcoal rot. 

## 5. Materials and Methods

### 5.1. Materials

Potato dextrose agar (PDA) dehydrated medium, glycine, β-alanine, lysine hydrochloride, ethylamine hydrochloride, and ethylenediamine dihydrochloride were purchased from Sigma-Aldrich, St. Louis, MO, USA. Kojic acid and orsellinic acid were purchased from Cayman Chemical Company, Ann Arbor, MI. Moniliformin was the gift of the late Dr. Ronald Vesonder, National Center for Agricultural Utilization Research, Peoria, IL, USA. (±)-Botryodiplodin was synthesized as described previously [40]. Unless otherwise indicated, all other chemicals and organic solvents were obtained from Fisher Scientific, Pittsburgh, PA, USA and used without purification. 

### 5.2. Fungal Strains, Media and Culture Conditions

*M. phaseolina* strains used in this study were isolated from soybean plants in Mississippi exhibiting symptoms of charcoal rot disease using the method of Mengistu et al. [5,46], or they were provided by colleagues at Mississippi State University [24]. Fungal cultures were maintained on potato dextrose agar medium at 25 °C, and stored on potato dextrose agar slants at −80 °C. Six isolates of *M. phaseolina* were selected for detailed study as examples of isolates in each of the following three red pigment production level categories (Figure 1): high: *Mp* 183 and *Mp* 230; moderate: *Mp* 264 and *Mp* 312; and none: *Mp* 009 and *Mp* 239. 

### 5.3. Extraction and Assay of (−)-Botryodiplodin and Pigment Produced on Agar Medium by Selected M. phaseolina Isolates

In-culture assays are typically conducted in agar medium. Since agar and other medium components interfere with assays for (−)-botryodiplodin and pigment, it was necessary to extract secreted substances from the agar medium in order to quantify them accurately. A collection of 39 *M. phaseolina* isolates drawn from the earlier 42-isolate collection were used to inoculate three plates of Czapek-Dox agar medium (2.0 g NaNO_3_, 1.0 g K_2_HPO_4_, 0.5 g KCl, 0.5 g MgSO_4_, 0.01 g FeSO_4_, 30 g sucrose, and 20 g agar in 1 L water) by placing a mycelium-covered 4 mm agar plug in the center of the plate. The inoculated plates were incubated at room temperature for seven days, then a flame-sterilized cork borer was used to cut two 4 mm agar plugs from the medium in each of the three culture dishes equally distant from the center of the *M. phaseolina* colony. One agar plug from each of the three *M. phaseolina* isolate plates was transferred to a separate well of a 96-well culture plate that was used for pigment extraction, and the other agar plug was transferred to a separate well of another 96-well culture plate that was used for (−)-botryodiplodin extraction. The 96-well plates were covered with parafilm, frozen overnight at −20 °C, then thawed at room temperature. For pigment extraction, 120 µL of water was added to each well containing an agar plug and the 96-well plate subjected to a second freeze-thaw cycle. The plates were thawed at room temperature, agitated for 5 min at room temperature on a plate rocker and a 100 µL aliquot of the supernatant liquid in each well transferred to another 96-well plate for direct measurement of pigment content as the absorbance at 520 nm in a plate reader (BioTek Instruments Synergy HT, Winooski, VT, USA). For (−)-botryodiplodin extraction, 120 µL of ethyl acetate was added to each well containing an agar plug. The agar plugs were squeezed into the ethyl acetate in each well with a glass rod. A 100 µL aliquot of the supernatant liquid in each well was transferred to an Eppendorf tube and dried under vacuum. The amount of (−)-botryodiplodin in the residue from each aliquot was measured by LC-MS using a LTQ XL Ion Trap Mass Spectrometer (Thermo Scientific, West Palm Beach, FL, USA) by reconstituting each sample in 1 mL ethyl acetate. The entire volume (1 mL) of each sample was then placed in individual vials and loaded onto an autosampler (Thermo Scientific, West Palm Beach, FL, USA). A 3.9 mm × 150 mm, 4 µm Nova-Pak C18 column (Waters Corporation, Milford, MA, USA) was used with elution in binary gradient mode at a flow rate of 500 µL/min using mobile phases containing ammonium acetate (5 mM) in acetic acid: water: methanol (eluent A: 1:89:10, *v*/*v*/*v*; eluent B: 1:2:97, *v*/*v*/*v*) as described below. 

### 5.4. (−)-Botryodiplodin Production by Selected M. phaseolina Isolates on Liquid Medium

Production of (−)-botryodiplodin and other known mycotoxins by the selected *M. phaseolina* isolates was also determined under widely used liquid culture conditions using LC-MS/MS. *M. phaseolina* isolates were cultured in 500 mL Erlenmeyer flasks containing 200 mL aliquots of autoclaved Czapek-Dox broth (CDB) prepared as described above, except without agar. Each flask was inoculated with five 5 mm mycelium-covered plugs from a 7-day-old PDA culture of each *M. phaseolina* isolate. Inoculated flasks were incubated for 10 days at 28 °C and 110 rpm in an Innova 40 Benchtop Incubator Shaker (New Brunswick Scientific Co., Inc., Edison, NY, USA). After incubation, the liquid cultures were initially filtered through cheesecloth to remove large biomass not able to pass through a fine filter, then cell-free culture filtrates of the *M. phaseolina* isolates were prepared by vacuum filtration in Nalgene Rapid-Flow sterile disposable filter units with 0.45 µm pore-size CN membrane filters (Thermo Fisher Scientific, Rochester, NY, USA) and freeze-dried. Dried cell-free culture filtrate powders were stored at −20 °C until shipped for chemical analysis studies. 

Samples were analyzed by LC-MS/MS as described by Sulyok et al. [47]. Briefly, a QTrap 5500 MS/MS system (Sciex, Foster City, CA, USA) containing a TurboV electrospray ionization source (Sciex, Foster City, CA, USA) served as detector for a UHPLC system (1290 series, Agilent Technologies, Waldbronn, Germany). Chromatographic separation used a Phenomenex Gemini C18 column (150 mm × 4.6 mm i.d. and 5 µm particle size) following a Phenomenex C18 security guard cartridge (4 mm × 3 mm i.d.) with a 5 µL injection volume and eluted in binary gradient mode at a flow rate of 1 mL/min. Elution used an initial 2 min of 100% mobile phase A (methanol/water/acetic acid 10:89:1 (*v*/*v*/*v*) containing 5 mM ammonium acetate), after which mobile phase B (methanol/water/acetic acid 97:2:1 (*v*/*v*/*v*) containing 5 mM ammonium acetate) was increased linearly to 50% over 3 min, then linearly increased to 100% over 9 min and B was held at 100% for 4 min. The column was re-equilibrated back to 100% A within 2.5 min between runs. Scheduled multiple reaction monitoring (sMRM) was used for analyte detection. Quantification of the analytes was based on external calibration using a serial dilution of a multi-component stock solution. Confirmation of a positive identification in this study was based on (i) the measured ion ratio corresponding within 30% to standard values given in SANTE/12089/2016 [48] or (ii) the retention time applying a stricter criterion of ±0.03 min. 

### 5.5. Glycine Concentration for Optimal Pigment Production

To determine the optimum amount of glycine for stimulation of pigment production by selected isolates of *M. phaseolina* growing on Czapek-Dox agar media, Petri dishes containing 20 mL of agar medium were flooded with 0.8 mL of sterile glycine solution for give final glycine concentrations of 0, 0.5, 1, 2, 4, 6, 8, 10, 12 and 14 mg/mL. A total of 30 plates were used with each isolate with 3 plates for each treatment glycine concentration. The plates at each glycine concentration were inoculated with a 4 mm Czapek-Dox agar medium disc covered with mycelium of one of the six selected *M. phaseolina* isolates. The plates were incubated at room temperature until the mycelial mat covered half or more of the agar surface. Three agar plugs were punched out and expelled into the wells of a 96-well plate. The plates were covered with parafilm and frozen overnight at −20 °C. After thawing, 100 µL of water was added to each well and the agar plugs were squeezed into the liquid in each well with a glass rod. The supernatant liquid (100 µL) in each well was transferred to the corresponding well of a clean 96-well plate. Red pigment formation was measured at OD_520_ in a microplate reader (BioTek Instruments Synergy HT, Winooski, VT, USA). 

### 5.6. Time Courses of Pigment and (−)-Botryodiplodin Production by Selected M. phaseolina Isolates

Triplicate cultures of the selected isolates of *M. phaseolina* were set up on 10 cm plates containing 20 mL of sterile Czapek-Dox agar medium with glycine supplementation (10 g/L) and inoculated with an agar plug from a seed culture plate of the selected *M. phaseolina* isolate. The cultures were incubated at room temperature for nine days. Agar plug samples from each culture were taken daily. The red pigment was extracted from each agar plug sample and read on a microplate reader. A similar procedure was carried out for the time course study of (−)-botryodiplodin production, except that no glycine was added to the media. (−)-Botryodiplodin was extracted with ethyl acetate and quantified by the procedure as stated above. The time courses of red pigment production and (−)-botryodiplodin production were plotted for each isolate. 

### 5.7. Stimulation of Pigment Production by Culture Medium Components

Petri plates (10 cm) containing 20 mL of Czapek-Dox agar medium with glycine (10 g/L) were prepared lacking one of the following components: NaNO_3_, K_2_HPO_4_, KCl, MgSO_4_, FeSO_4_ or sucrose, but including glycine, agar and water. Triplicate plates were inoculated by placing 5 mm from the plate edge a 4 mm agar medium plug, mycelium-side down, completely covered with one of the following *M. phaseolina* isolates growing on complete Czapek-Dox agar medium: *Mp* 183, *Mp* 230, *Mp* 264 and *Mp* 312. A Czapek-Dox agar medium plug this size contains a sufficient amount of the medium component missing from the plate to support hyphal growth across the entire agar surface. On the other side of the plate a 4 mm diameter well was cut in the agar medium with a flame-sterilized cork borer 5 mm from the edge. Inoculated plates were incubated at room temperature until hyphal growth was half way across the plate, when a sterile solution of the Czapek-Dox medium component that the plate was deficient in was added to the well as 200 μL of 50 times the normal Czapek-Dox medium concentration on two consecutive days. The agar medium between that well and the inoculating plug was examined daily for pigment formation, microsclerotia formation and intense hyphal branching visible on the agar surface when viewed under a microscope. 

Pigment produced in the presence of a range of sucrose concentrations in Czapek-Dox agar medium was determined by extracting it from agar plugs (4 mm) cut from plates covered with mycelium of four selected *M. phaseolina* isolates, *Mp* 183, *Mp* 230, *Mp* 264 and *Mp* 312. These isolates were used to inoculate plates with Czapek-Dox agar medium minus sucrose, which were incubated at room temperature until mycelium had spread extensively across the agar surface. Agar plugs (0.4 cm) were cut from Czapek-Dox agar medium minus sucrose where the surface was covered with mycelium, but as far as possible from the inoculating plug, and used to inoculate 10 cm Petri plates containing 20 mL of Czapek-Dox agar medium minus sucrose plus glycine (10 g/L) plus a range of sucrose concentrations from 0 to 30 g/L (100% of that used in complete Czapek-Dox agar medium). The inoculated plates were incubated at room temperature until mycelium covered the agar surface of all of the plates, including 0% sucrose. Pigment production in response to added sucrose was quantitated by cutting three 3 mm agar plugs 5 mm from the plate edge distributed around the plate. The agar plugs were placed on edge in the wells of 96-well trays, covered with 175 μL of water, subjected to freeze-thaw, rocked for 5 min, then 100 μL of each extract solution transferred to the corresponding wells of 96-well trays and absorbances read at 520 nm in a microplate reader (BioTek Instruments Synergy HT, Winooski, VT, USA). 

### 5.8. Pigment Formation by Reaction of (±)-Botryodiplodin with Amines in Solution

Serial dilutions of amine solutions (100 µL of the concentration given in the legend to Figure 5) were carried out in water in triplicate in the wells of 96-well microtiter trays and 100 µL of (±)-botryodiplodin (1.0 mg/mL) added to each well. The wells of the tray were covered with parafilm, incubated overnight at room temperature and scored visually for color formation. 

### 5.9. UV-Vis Absorption Spectra of Pigments Formed in Agar Medium 

Petri plates (10 cm) were prepared in triplicate with 20 mL of Czapek-Dox agar medium lacking sucrose and supplemented with 10 g/L of glycine, β-alanine, lysine hydrochloride, ethylamine hydrochloride, or ethylenediamine dihydrochloride. Flame-sterilized cork borers were used to cut wells in the agar medium of each plate located at 2 o’clock (4 mm diameter) and 5 o’clock (3 mm diameter) at 5 mm from the edge. Plates were inoculated in triplicate by placing mycelium-side down at 9 o’clock at 5 mm from the edge of the medium surface a 4 mm diameter agar medium plug completely covered with mycelium of one of the following *M. phaseolina* isolates growing on complete Czapek-Dox agar medium: *Mp* 183, *Mp* 230, *Mp* 264 and *Mp* 312. Inoculated plates were incubated at room temperature until hyphal growth was halfway across the plate, when 200 μL of a sterile solution of sucrose (200 µL of 0.5 g/mL) was added to the 2 o’clock well on two consecutive days. When the agar medium between the sucrose well and the inoculating plug reached near maximal pigment formation, (±)-botryodiplodin (60 µL of 1 mg/mL) was placed in the 0.3 cm well. The plates incubated at room temperature overnight, during which time substantial red pigment formation occurred in the agar layer around the well receiving (±)-botryodiplodin. For each plate, three 3 mm plugs of pigment-containing agar were cut with a cork borer around the (±)-botryodiplodin well and in the pigment-formation zone between the inoculum plug and the sucrose well. The agar plugs from each source were combined into a sterile tube, covered with 1.2 mL water, placed in a freezer overnight, then thawed, a 1.0 mL aliquot of the supernatant drawn off and the UV-Vis absorbance spectra determined for each aliquot from 200 to 800 nm on a Beckman Coulter DU *730* UV/Vis spectrophotometer instrument (Brea, CA, USA). 

### 5.10. Statistical Analysis

The data were subjected to analysis of variance (ANOVA), Pearson correlation coefficient, regression analysis or Spearman’s rank correlation coefficient using the statistical package in Microsoft Excel. Each mean was calculated from triplicate values. *p* < 0.05 was considered significant. 

## Figures and Tables

**Table 1:** Production of known mycotoxins and other metabolites by selected *Macrophomina phaseolina* isolates determined by LC-MS/MS.

*M. phaseolina* Metabolite (mg/L)	*M. phaseolina* 230	*M. phaseolina* 183	*M. phaseolina* 312	*M. phaseolina* 264	*M. phaseolina* 009	*M. phaseolina* 239
(−)-Botryodiplodin	34.0	64.7	56.6	44.0	0.0	0.0
Moniliformin	0.006	0.045	0.031	0.007	0.082	0.0
Kojic acid	0.142	0.044	0.109	0.102	0.0	0.0
Orsellinic acid	2.86	1.90	1.86	0.722	0.0	0.563
Cordycepin	0.0	0.0	0.0	0.0	0.001	0.002
cyclo(L-Pro-L-Tyr)	0.0	0.0	0.0	0.0	0.095	0.022
cyclo(L-Pro-L-Val)	0.0	0.0	0.0	0.0	0.134	0.067
Gliocladic acid	0.0	0.018	0.0	0.0	0.0	0.0
Dihydrocitrinone	0.0	0.0	0.0	0.0	0.001	0.0

## Data Availability

Not applicable.
